# The Clarence Memorial Wing at St. Mary's Hospital

**Published:** 1892-05-28

**Authors:** 


### FESTIVALS AND DINNERS.

### THE CLARENCE MEMORIAL WING AT ST. MARY'S HOSPITAL.

Ever since its foundation St. Mary's Hospital has been struggling with the utter inadequacy of its accommodation to the needs of the large and growing district to which it ministers. As long ago as 1841, when the district and population were only about half what they are at present, it was thought necessary that the institution should contain 380 beds, but at that time sufficient funds were not forthcoming to carry out the wishes of the founders, and for many years the charity had to be content with less than half this accommodation, viz., 150 beds. Plans at the time were, however, prepared for a hospital as large as the grounds would admit of, and gradually, bit by bit, the institution has been added to, until at the present time it is as large as the space at the disposal of the Managers would allow. The strain upon its resources, however, continued so great that further room was made by providing sleeping accommodation for the nursing and other staff outside the hospital walls. This has of necessity proved but a temporary alleviation, and so it became absolutely essential that more property should be acquired and the hospital still further extended. Another disadvantage which St. Mary's laboured under, that of being situated out of the main thoroughfare, pointed to the direction in which such extension should be made, and the Managers set about trying to buy the property which lay between the hospital and Praed Street. For some time the difficulties in the way proved insurmountable, but perseverance has at length overcome them, with the result that everything is now ready for building operations.

On the site which has after so much trouble been at last obtained it is proposed to erect five blocks, to contain (1) a nurses' home; (2) a new out-patients' department, the old one being ill-ventilated, badly heated, and far too small for the great number of out-patients frequenting it; (3) special wards, including wards for lying-in cases; (4) a new administration block; and (5) a residential college for the students attending the school. The old administrative department on the ground floor of the present building will be converted into additional accident wards, while the present special wards will be set free for ordinary medical and surgical cases. These alterations and additions will give the Managers, within the boundaries of the hospital site, a hospital containing 380 beds, with accommodation for all its nursing staff, and a medical school and college, thus making the institution complete and self-contained, and, at the same time, more able to cope with the treatment of the sick and suffering of Paddington and the surrounding districts.

The hospital has always enjoyed a close connection with our Royal Family. At its inception the foundation-stone was laid by the late Prince Consort, Her Majesty the Queen is its Patron, the Marchioness of Lorne opened the "Mary Stanford Wing" in 1884, Prince George of Wales is its President, and the Prince and Princess of Wales, besides consenting that the new extension should be named as a memorial to the Duke of Clarence, are to lay the foundation-stone in October next.

Having thus shown the needs of the hospital, we come to the most important point of all—the cost. Already the purchase of the property has mounted up to £45,000, and another £55,000 will have to be spent on the buildings, making in all £100,000 to be raised before the work can be completed. We do not think, however, that the authorities of the institution need despair about the possibility of raising such a large sum of money, if we may judge from the excellent result of the Festival Dinner, held at the Hotel Metropole on the 20th inst., when, through the herculean exertions of all concerned, no less a sum than £10,600 was raised towards this object. Of this sum Mr. G. P. Field, the Chairman of the Dinner Committee, succeeded in collecting £3,208, and we congratulate both Mr. Field and Mr. Thomas Ryan, the Secretary, on the result which has been achieved. The utmost enthusiasm prevailed on that occasion, and if those present only show half the sympathy and energy in the cause of the hospital outside the walls of the Metropole that they displayed at the dinner the necessary funds will be to hand in a very short time.

Mr. J. D. Allcroft, a keen and constant supporter of the institution, presided at the dinner in the unavoidable absence of H.R.H. the Duke of Cambridge. In proposing the toast, "Prosperity to St. Mary's Hospital," Mr. Allcroft said that the hospital was built in 1845, and at that time was probably equal to the requirements of the neighbourhood, but since that year the surrounding district, as they all knew, had grown so considerably, and the population had so increased that further extension was urgently needed. That such extension was an absolute necessity was shown by the fact that should a case not be able to get admission to St. Mary's, it had to be taken either to the other side of Hyde Park to St. George's Hospital, or as far west as Berners Street to the Middlesex Hospital, the two nearest general hospitals. What an amount of unnecessary suffering that entailed he need not tell them. The new wing, for which they had bought the property, would extend to Praed Street, and would contain additional special wards, lying-in wards, a new out-patients' department, a nurses' home, and a residential college for medical officers and students. As to the first and second, he was very much in favour of concentrating the work of the special hospitals in the general hospitals. This was the first time in recent years that a general hospital proposed to have a ward for lying-in cases. St. Mary's, during the past year, had treated a thousand maternity cases, and the need for such a ward was very clear and undoubted. A nurses' home, too, was a great necessity, when the disadvantage of the large staff of nurses necessary for the patients having to sleep out of the hospital was taken into consideration. It was desirable also that the students should be resident at the hospital, as they would then be close to their work, and under the observation of the management. One great advantage which the hospital possessed was the fact that they had the sympathy, support, and encouragement of many members of the Royal family. (Applause). The Queen was their Patron and had contributed towards their building fund, the Prince and Princess of Wales had kindly consented that the wing should be called Clarence Wing, in memory of their son whom all had deeply mourned, and their remaining son, Prince George, had agreed to become the President of the hospital. (Applause.) During the year 1891 the in-patients had numbered 3,922; out-patients, 18,900; and the casualities 10,478, making the total number of persons who had been benefited 33,300. (Applause.)

